# Supplementary material for: Alcohol withdrawal syndrome in ICU patients: Clinical features, management, and outcome predictors
Source: PLoS One. 2021 Dec 20;16(12):e0261443. doi: 10.1371/journal.pone.0261443 (PMC8687554; doi:10.1371/journal.pone.0261443)
Supplement: S1 Fig — (DOCX) [file pone.0261443.s001.docx]

**S1 Fig. Diagnostic criteria for alcohol withdrawal syndrome according to the Diagnostic and Statistical Manual of Mental Disorders (Fifth Edition) (DSM-5)**

All 4 must be present to diagnose alcohol withdrawal.

A. Cessation of (or reduction in) alcohol use that has been heavy and prolonged

B. Two (or more) of the following, developing within several hours to a few days after cessation of (or reduction in) alcohol use described in Criterion A:

1. Autonomic hyperactivity (eg, sweating or pulse rate greater than 100 bpm)

2. Increased hand tremor

3. Insomnia

4. Nausea or vomiting

5. Transient visual, tactile, or auditory hallucinations or illusions

6. Psychomotor agitation

7. Anxiety

8. Generalized tonic-clonic seizures

C. The signs and symptoms in Criterion B cause clinically significant distress or impairment in social, occupational, or other important areas of functioning

D. The signs or symptoms are not attributable to another medical condition and are not better explained by another mental disorder, including intoxication or withdrawal from another substance
