# Supplementary material for: Alcohol withdrawal syndrome in ICU patients: Clinical features, management, and outcome predictors
Source: PLoS One. 2021 Dec 20;16(12):e0261443. doi: 10.1371/journal.pone.0261443 (PMC8687554; doi:10.1371/journal.pone.0261443)
Supplement: S1 Table — (DOCX) [file pone.0261443.s002.docx]

**S1 Table. Comparison of ICU patients with and without AWS during the study period**

| **Variable** | **Patients**  **with AWS**  **(n = 204)** | **Patients without AWS**  **(n =5437)** | ***P* value** |
| --- | --- | --- | --- |
| **Demographics** |  |  |  |
| Age, median [IQR], years | 53 [46-60] | 60 [45-70] | 0.002 |
| **SAPS II**, median [IQR], years | 24 [16-34] | 35 [24-52] | 0.001 |
| **Outcome** |  |  |  |
| ICU LOS, median [IQR], days | 6 [4-10.3] | 3 [2-6] | <0.001 |
| ICU LOS≥7days, n (%) | 90 (44) | 1095 (20.1) | <0.001 |
| Hospital mortality, n (%) | 16 (7.8) | 786 (14.5) | 0.008 |
| **Complicated hospital stay** |  |  |  |
| ICU LOS ≥7 days or hospital death, n (%) | 98 (48) | 1685 (31) | <0.001 |

AWS: alcohol withdrawal syndrome; ICU: intensive care unit; IQR: interquartile range; LOS: length of stay; SAPS II: Simplified Acute Physiology Score, version II
