# Supplementary material for: Alcohol withdrawal syndrome in ICU patients: Clinical features, management, and outcome predictors
Source: PLoS One. 2021 Dec 20;16(12):e0261443. doi: 10.1371/journal.pone.0261443 (PMC8687554; doi:10.1371/journal.pone.0261443)
Supplement: S2 Table — (DOCX) [file pone.0261443.s003.docx]

**S2 Table. Logistic regression analyses for factors associated with complicated hospital stay among the 5,641 patients admitted to the ICU during the study period.**

| **Factors** | **Multivariable analysis** | |
| --- | --- | --- |
|  | **OR (95%CI)** | ***P* value** |
| **Age (per year)** | 0.99 (0.99-1.00) | 0.368 |
| **SAPS II (per point)** | 1.06 (1.05-1.06) | <0.001 |
| **Alcohol withdrawal syndrome** | 3.53 (2.60-4.81) | <0.001 |

SAPS II: Simplified Acute Physiology Score, version II

Candidate predictors were: Age, SAPS II, and alcohol withdrawal syndrome.
